# Genetic diversity of *Streptomyces* species causing potato common scab in northern China

**DOI:** 10.1128/spectrum.03050-25

**Published:** 2026-03-19

**Authors:** Yuchen Li, Chi Li, Jianjun Hao, Jianping Zhang, Liwei Wang, Qi Wang, Pingping Sun, Wenbing Zhang, Xiaoyu Zhang

**Affiliations:** 1 Key Laboratory of Biopesticide Creation and Resource Utilization in Inner Mongolia Autonomous Region, College of Horticulture and Plant Protection, Inner Mongolia Agricultural University 117454 , Hohhot, China; 2 State Key Laboratory of Agricultural and Forestry Biosecurity, MARA Key Lab of Surveillance and Management for Plant Quarantine Pests, College of Plant Protection, China Agricultural University, Beijing, China; 3 Guangdong Provincial Key Laboratory of Biotechnology for Plant Development, School of Life Sciences, South China Normal University, Guangzhou, China; 4 School of Food and Agriculture, University of Maine 115975, Orono, Maine, USA; 5 Institute of Plant Protection, Inner Mongolia Academy of Agriculture and Animal Husbandry Sciences 232813 , Hohhot, China; University of Georgia, Athens, Georgia, USA

**Keywords:** *Streptomyces galilaeus*, pathogenicity, genetic diversity, txtAB, MLSA

## Abstract

Potato common scab (PCS) is caused by various *Streptomyces* spp., which seriously affect the quality and economic value of potatoes. Different species of pathogens may affect disease control strategies due to their biological characteristics. To determine pathogen composition in the major potato production area, northern China, potato tubers with various types of PCS symptoms were collected from 18 counties. From 57 collected samples, 159 bacterial isolates of *Streptomyces* spp. were obtained on oatmeal agar. Pathogenicity of these isolates was examined on tuber disks and potted plants of potato, resulting in 42 isolates being confirmed as pathogenic and causing typical scabby symptoms. To confirm pathogenicity determinants, the *txtAB*, *tomA*, and *nec1* genes of these pathogens were detected using polymerase chain reaction (PCR). Pathogenic isolates were also identified by the combination of morphological, physiological, and biochemical characteristics, BOX-PCR, *16S* rDNA, and multilocus sequence analysis. Among the 42 pathogenic *Streptomyces* isolates, 36 (85.7% of total isolates) were identified as *Streptomyces galilaeus*, and 6 (14.3%) as *Streptomyces scabies*. The genome of *S. galilaeu*s isolates from the predominant group was resequenced, yielding six distinct genetic clusters. The Shangdu population exhibited the most complex genetic structure. The dominance and genetic diversity of *S. galilaeus* revealed in this study provide a critical foundation for breeding resistant potato varieties and developing targeted control measures tailored to the agroecological conditions of northern China.

**IMPORTANCE:**

Potato common scab (PCS) caused by pathogenic *Streptomyces* seriously affects the quality and economic value of potato, especially in northern China. The species of *Streptomyces* were closely related to their geographical distribution. We determined that *Streptomyces galilaeus* is dominant in this area, rather than *Streptomyces scabies*, which is widely distributed. This finding emphasizes the importance of developing effective prevention and control strategies for the region. In addition, genome resequencing of *S. galilaeus* revealed significant genetic diversity, with the Shangdu population displaying the most complex structure. These results provide important information for further understanding the population structure and distribution of PCS pathogens.

## INTRODUCTION

Potato common scab (PCS) widely occurs in most potato-producing areas (1). This disease has progressively increased in China as potato production is extensively growing, which is especially a problem in areas with an arid climate and continuous cropping of potatoes, such as in northern China. Common scab in this area is a major production constraint, which can reach as high as 100% of PCS (2). However, the species and distribution of pathogens causing PCS in this region are not well defined.

PCS affects the early growth and development of potato plants, delaying seedling emergence and sometimes reducing yield (3). However, the greatest impact is on the cosmetic appearance of the tubers, which leads to their downgrading. Symptoms include superficial, raised, and pitted lesions. The development of different types of scabby spots may vary based on potato varieties, environmental conditions, isolate types, and the toxin content of the pathogens (4).

The main pathogenic determinant of *Streptomyces* is several thaxtomins. These compounds belong to a family of toxic metabolites possessing a cyclic dipeptide (5), in which thaxtomin A is the main toxin component. The non-ribosomal peptide synthetases, specifically TxtA and TxtB, are involved in the production of thaxtomin A. Therefore, the *txtAB* gene is the decisive gene for whether an isolate is pathogenic (6).

The identification of *Streptomyces* has always been a challenging task. Since the 1960s, the implementation of the International Streptomyces Project (ISP) and the emergence of various chemical classification methods have made great progress in the classification of *Streptomyces*, followed by the application of molecular biology methods in the 1980s, especially the emergence of sequence analysis of 16S rDNA, enabling *Streptomyces* spp. to be better distinguished. Moreover, DNA-DNA hybridization, multilocus sequence analysis (MLSA), DNA fingerprinting, and average nucleotide identity have been developed for improved classifications (6, 7).

 Dozens of *Streptomyces* spp. are pathogenic to potatoes. *Streptomyces scabies*, *S. acidiscabies*, and *S. turgidiscabies* were first reported, followed by *S. aureofaciens*, *S. europaeiscabiei*, *S. luridiscabiei*, *S. niveiscabiei*, *S. puniciscabiei*, *S. reticuliscabiei* and *S. stelliscabiei*, *S. caviscabies*, *Streptomyces galilaeus*, *S. alkaliscabies*, and *S. albidoflavus* (6, 8–10). Nonetheless, new pathogenic *Streptomyces* are constantly being reported (11–13).

The taxonomy of *Streptomyces* is related to their geographical distribution. For example, the predominant species are *S. scabies*, *S. turgidiscabies*, and *S. europaeiscabiei* in Europe (14); *S. scabies*, *S. acidiscabies*, *S. europaeiscabiei*, and *S. stelliscabiei* in the United States (6, 12); *S. scabies*, *S. acidiscabies*, and *S. caviscabies* in Canada (15); *S. scabies* and *S. stelliscabiei* in Africa (16); *S. scabies*, *S. niveiscabiei*, *S. acidiscabies*, *S. puniciscabiei*, *S. turgidiscabies*, and *S. luridiscabiei* in Korea (17); *S. cinereus*, *S. collinus*, and *S. longisporoflavus* in India (18); and *S. scabies*, *S. turgidiscabies*, and *S. acidiscabies* in Japan (19).

Single-nucleotide polymorphisms (SNPs) are one of the most prevalent forms of genetic variation (20). Due to their extensive distribution and significant conservation across the genome, SNPs can be accurately mapped and leveraged as high-resolution genetic markers. This precision has led to the successful application of SNP technology in species classification within microbiology, notably in distinguishing between strains of bacteria such as *Brucella* and *Staphylococcus aureus* (21, 22). As a result, SNPs have become powerful tools for identifying bacterial strains, exploring their genetic diversity, and facilitating phylogenetic analysis.

The distribution of PCS pathogens in China also exhibits distinct regional characteristics. Preliminary surveys have indicated a pathogenic population in China comprising *S. scabies*, *S. acidiscabies*, *S. galilaeus*, and others (23, 24). However, there is still a lack of systematic understanding of the pathogen population structure, dominant species, and their genetic diversity in the major potato-growing regions in northern China. Based on this, this study aims to clarify the species composition and dominant populations of PCS pathogens in northern China through MLSA; evaluate their pathogenicity through the detection of virulence genes and pathogenicity tests; and analyze the population genetic structure of the dominant species using SNP markers, providing a theoretical basis for the precise prevention and control of regional diseases.

## MATERIALS AND METHODS

### Pathogen isolation

Potato tubers showing PCS symptoms were collected from various field locations in northern China during 2015–2018. The tubers were washed with tap water to remove soil. Necrotic lesions were dissected and cut into a size of around 0.5 cm^2^ in size and 5 mm. The cut tissues were treated in 75% ethanol for 20 s, washed three times with sterile water, and thoroughly ground using a sterile mortar and pestle. One milliliter of the suspension was subjected to a 10-fold serial dilution, generating 10^−1^, 10^−2^, and 10^−3^ dilutions. An aliquot of 100 μL of each dilution was dispensed onto an oatmeal agar (OMA) plate and incubated at 28°C for 7 days. A single colony was picked and transferred to a new plate for purification, which was stored at 4°C for later use.

### Pathogenicity assays

#### Inoculation on potato tuber disks

Healthy potato “Desiree” tubers were washed with tap water and peeled to remove skins. The tuber was disinfected by soaking in 75% alcohol for 30 s. They were cut into disks with a diameter of 1.5 cm and a thickness of 2 mm by using a cork borer and placed in a Petri dish with sterile filter paper placed on the bottom. The experiment was repeated three times, with five disks placed in each plate. A culture plug (0.5 cm in diameter) of a 7-day-old *Streptomyces* isolate grown on OMA plates was placed at the center of potato disks. A plug of OMA without bacteria and the PS1 standard strain of *S. galilaeus* preserved in the laboratory was used as negative and positive controls, respectively. The plates were incubated in the dark at 25°C and evaluated for lesions 5 days after incubation. The area of the lesion was measured perpendicularly.

#### Inoculation on potted plants

Healthy potato tubers (Desiree) were soaked in 0.6% NaClO for 10 min for surface disinfection and then seeded into a pot with 20 cm-diameter opening and 15 cm height which was filled with sterilized vermiculite and potting soil mixed at a 1:1 ratio, five pots per isolate, with four tuber pieces in each pot. Bacteria cultured on OMA plates for 10 days were blended in a tissue grinder, diluted to 10^7^ CFU/mL with distilled water, of which 100 mL was inoculated into each soil-filled pot. Uninoculated agar and PS1 standard strain of *S. galilaeus* preserved in the laboratory were used as negative and positive controls, respectively. The inoculation was conducted 30 days after sowing. The pots were placed in the open space and maintained using a standard practice.

Potato tubers were harvested 100 days after planting, and tuber disease was evaluated. Disease severity was expressed as the following rating scale based on the percentage of lesion of total tuber surface: 0 (no lesion), 1 (more than 1% less than 5%), 2 (more than 5% less than 25%), 3 (more than 25% less than 50%), 4 (more than 50% less than 75%), and 5 (more than 75%). Incidence of PCS was calculated as the number of diseased tubers divided by the total evaluated number of tubers × 100%:


Diseaseindex=100×sum(Ni)/totalnumberofevaluatedtubers×5

where *i* is the disease scale (from 0 to 5), and *N* is the number of diseased tubers at level *i*.

### Pathogenicity determination and pathogenic gene detection

Genomic DNA of bacterial isolates was extracted using the TIANamp Bacteria DNA Kit (Tiangen Biotech Co., Ltd, Beijing, China). Polymerase chain reaction (PCR) was conducted using the extracted DNA as a template. The primer pairs required for PCR were TxtAB1: 5′-CCACCAGGACCTGCTCTTC-3′ and TxtAB2: 5′-TCGAGTGGACCTCACAGATG-3′ (6), Nf: 5′-ATGAGCGCGAACGGAAGCCCCGGA-3′ and Nr: 5′-GCAGGTCGTCACGAAGGATCG-3′ (25), and TomF: 5′-GAGGCGTTGGTGGAGTTCTA-3′ and TomR: 5′-TTGGGGTTGTACTCCTCGTC-3′ (6). The PCR reaction mixture (total volume 50 μL) included 34 μL ddH_2_O, 5 μL 10× PCR buffer, 4 μL dNTPS (2.5 mmol/L), 2 μL forward primer (10 pmol/µL), 2 μL reverse primer (10 pmol/µL), 1 μL DNA (80 ng/µL), and 0.5 μL Taq DNA polymerase (5 U/µL). The setting of the thermal cycler was pre-denaturation at 94°C for 5 min, followed by 30 cycles of denaturing at 94°C for 1 min, annealing at 53°C for *txtAB*, 62°C for *nec1*, and 53°C for *tomA* for 1 min, and extension at 72°C for 1 min. A final reaction at 72°C for 10 min was applied. PCR products of the target gene segment were detected by electrophoresis on a 1% agarose gel. PCR products were purified using the Gel Extraction Kit (Omega Bio-Tek Co., Ltd, Guangzhou, China).

### Pathogen identification

Two groups of *Streptomyces* with representative morphological characteristics were studied. The bacterium was cultured on a series of identification media for *Streptomyces* designated by the International Streptomyces Project as ISP1, ISP2, ISP3, ISP4, ISP5, ISP6, and ISP7 (26) at 28°C. Bacterial growth was examined on the 7th, 14th, and 21st days of incubation. Morphological characteristics were observed, including colony color, formation of aerial hyphae, and the presence of soluble pigments. Specific morphologies, such as spore silk and spores, were photographed using an optical microscope. For characterizing the physiology and biochemistry of pathogens, methods of Berger handbook of bacteria identification (27) and the Handbook of *Streptomyces* identification (28) were employed. The physiological and biochemical indicators of pathogens, carbon source, nitrogen source utilization, sensitivity measurement, melanin, H_2_S production, starch hydrolysis, gelatin liquefaction, salt tolerance, and pH sensitivity were measured.

BOX-PCR fingerprinting was performed using the primer BOXAIR, with sequence 5′-CTACGGCAAGGCGACGCTGACG-3′ (29). The PCR reaction system was described as above, and the annealing temperature was 50°C. The bacteria were also identified by analyzing the DNA sequences of *16S* rDNA and multiloci. PCR was conducted using the primer pair PrimeF: 5′-CATTCACGGAGAGTTTGATCC-3′ and PrimeR: 5′-AGAAAGGAGGTGATCCAGCC-3′ (11), which target the *16S* rDNA gene. Genes including *atpD*, *rpoB*, and *recA* were amplified, with primer pairs atpDF: 5′-ACCAAGGGCAAGGTGTTCAA-3′ and atpDR: 5′-GCCGGGTAGATGCCCTTCTC-3′, rpoBF1: 5′-TTCATGGACCAGAACAACC-3′ and rpoBR1: 5′-CGTAGTTGTGACCCTCCC-3′, and recAF: 5′-ACAGATTGAACGGCAATTCG-3′ and recAR: 5′-ACCTTGTTCTTGACCACCTT-3′ (30). The PCR reaction system was described above, and the annealing temperature was 60°C for *16S* rDNA, 57°C for *atpD*, 65°C for *rpoB*, and 48°C for *recA*. PCR products were sequenced at Sangon Biotech (Shanghai) Co., Ltd. (Shanghai, China). The obtained 16S rDNA sequences were compared with those in the GenBank database using BLAST (https://www.ncbi.nlm.nih.gov). A representative concatenated sequence was generated for the isolate by assembling the *16S* rDNA, *atpD*, *rpoB*, and *recA* gene segments from the 10 isolates (31) using a sequence analysis tool (http://www.novopro.cn/tools/combine_fasta.html). A phylogenetic tree was constructed using MEGA7 (MEGA International, New York, USA).

### Genetic diversity analysis of *S. galilaeus*

#### Resequencing and SNP calling

Genomic DNA was extracted as previously described. Sequencing libraries were constructed following the manufacturer’s standard protocol. After passing quality control, the libraries were sequenced on an Illumina NovaSeq 6000 platform to generate 150 bp paired-end reads. The raw sequencing data were then subjected to quality assessment and filtering to obtain high-quality clean reads for subsequent bioinformatic analysis. Variant identification (including SNPs and small insertions/deletions) was performed following a resequencing approach based on the reference genome *S. galilaeus* ATCC 14969 (GenBank: FN554889.1). Briefly, clean reads from each strain were aligned to the reference genome using BWA-MEM. Duplicate reads were then marked and removed using the Picard toolkit. The GATK HaplotypeCaller (32) was employed for variant calling. The resulting variants were filtered to ensure high quality.

#### Phylogenetic and population genetic analysis

A core-genome SNP alignment, derived from the resequencing data of all 36 *S*. *galilaeus* strains, was used for downstream analyses. A maximum-likelihood phylogenetic tree was constructed from this alignment using MEGA 7, with branch support assessed by 1,000 bootstrap replicates. Population structure was inferred using ADMIXTURE (33) by testing potential genetic subgroups (*K*) from 1 to 10. The optimal *K* value was selected based on the lowest cross-validation error. Principal component analysis was performed using the EIGENSOFT (34) package to visualize genetic clustering.

### Data analysis

Statistical analysis was performed using the SPSS software (IBM SPSS Statistics, version 20; International Business Machines Corporation, New York, USA). Data were collected in triplicate and subjected to one-way analysis of variance. Means were separated by Tukey’s multiple range test at a significance level of *P* < 0.05.

## RESULTS

### Sample collection and isolation of PCS pathogen

**Fig 1:**
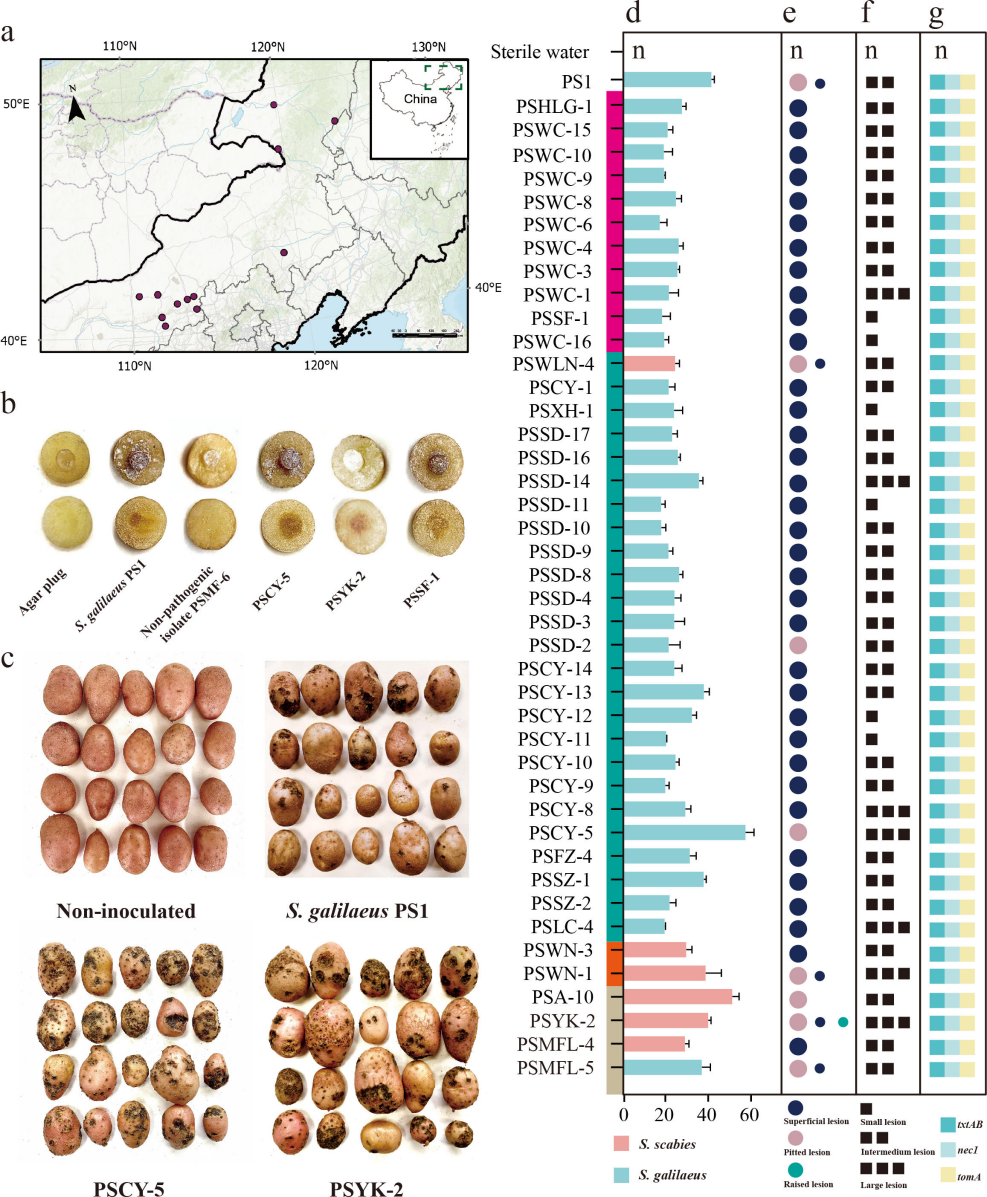
Pathogenicity of *Streptomyces* from northern China. (**a**) Sampling locations for 18 counties. The base map was obtained from the National Catalog Service for Geographic Information (Tianditu) and visualized using ArcGIS Pro. (**b and f**) Potato tuber disks, observed 5 days after inoculation. Lesion severity was scored on a 0–3 scale. (**c**) Typical common scab symptoms on tubers from potted potato plants inoculated with *Streptomyces* isolates. (**d**) Disease severity index of potted plants inoculated with different isolates. Bars represent mean ± SD (*n* = 5). (**e**) Classification of lesion types observed on tubers from pot experiments: superficial, raised, and pitted. (**g**) Detection of pathogenicity genes (*txtAB*, *tomA*, and *nec1*) by PCR in selected pathogenic isolates.

A total of 57 potato scab samples were collected from 18 counties of China (Fig. 1a), and 159 isolates of *Streptomyces* were obtained (Table 1; Table S1). The symptoms of potato scab lesions were divided into three types (Fig. S1): pitted, raised, and superficial lesions. The types of potato lesions collected in Hulunbuir (Hailar, Yakeshi, Zhalantun, and Arong) had all three mentioned types, including pitted, raised, and superficial lesion types. However, the samples collected in Chifeng (Keshiketeng and Wengniute) had superficial lesions; Ulan Qab (Liangcheng, Siziwangqi, Fengzhen, Chahar Right Middle, Shangdu, Xinghe, Huade, Chahar Right Back, and Jining) had superficial and pitted types; and Hohhot (Wuchuan and Hollinger) had superficial and raised types.

**TABLE 1:** Potato samples for *Streptomyces* isolation

City/county	Symptom type	Number of samples	Number of isolates
Hailar	Superficial	5	13
Yakeshi	Raised	2	10
Zhalantun	Pitted	2	4
Arong	Raised	2	4
Aershan		4	17
Keshiketeng	Superficial	2	5
Wengniute		4	14
Liangcheng		2	5
Siziwangqi		2	4
Fengzhen		3	7
Chahar Right Middle	Superficial, pitted	6	20
Shangdu	Superficial, pitted	7	20
Xinghe	Superficial	3	4
Huade		2	3
Chahar Right Back	Superficial	2	4
Jining	Superficial, pitted	2	5
Wuchuan	Superficial	5	18
Hollinger	Raised	2	2
Total		57	159

### Forty-two pathogenicity isolates harboring key pathogenic genes

The pathogenicity of 159 isolates of *Streptomyces* was determined on potato tuber disks. After 5 days of inoculation, 42 *Streptomyces* isolates produced noticeable lesions on potato tuber disks, indicating their pathogenicity. The remaining isolates did not produce symptoms, so they were classified as non-pathogenic *Streptomyces* (Fig. 1b and f).

The 42 isolates showing pathogenicity as mentioned above were used for pot inoculation assay (Table 2; Fig. 1c through e). All of the isolates caused typical scab symptoms on potato tubers. The virulence was significantly different among the 42 pathogenicity isolates. The most virulent isolate was PSCY-5, with a disease index of 56.98 and an incidence rate of 100% (all inoculated tubers developed symptoms), followed by PSA-10, with a disease index of 50.68 and an incidence of 97.3%. Both isolates showed pitted lesions. To follow Koch’s rule, we reisolated strains (PSYK-2 and PSCY-5) from potato tubers that had identical morphological characteristics compared with the original isolates (Fig. S4), consistent with the molecular identification of the original isolate.

**TABLE 2:** Physiological and biochemical characteristics of bacterial isolates

Nutrient source^*a*^	Group I	Group II	Nutrient source	Group I	Group II
L-arabinose	−	−	Histidine	+	+
D-fructose	−	−	Melanin production	+	+
D-xylose	−	−	Starch hydrolysis	+	+
D-mannitol	+	+	pH 4.0	−	−
I-inositol	+	+	pH 5.0	+	+
L-rhamnose	−	+	pH 6.0	+	+
D-glucose	+	+	3% NaCl	+	+
Sucrose	−	+	5% NaCl	−	−
Raffinose	+	+	10% NaCl	−	−
D-galactose	−	−	H_2_S generation	+	+
D-sorbitol	+	+	Gelatin liquefaction	+	+
Potassium nitrate	+	+	Crystal violet	−	−
Ammonium sulfate	+	+	Phenol	−	−
Sodium nitrate	+	+	Penicillin	−	−
L-methionine	+	+	Streptomycin	−	−
L-hydroxyproline	+	+			

^*a*^ +, positive reaction; −, negative reaction.

PCR products corresponding to *txtAB*, *tomA*, and *nec1* genes were detected by agarose gel electrophoresis and displayed with sizes of approximately 385, 700, and 392 bp, respectively (Fig. S5). All 42 isolates that were confirmed as pathogenic by inoculation assays as described above contained the *txtAB*, *nec1*, and *tomA* genes, which were not found in the non-pathogenic isolates (Fig. 1g).

### Morphological and molecular identification delineated two pathogenic species

**Fig 2:**
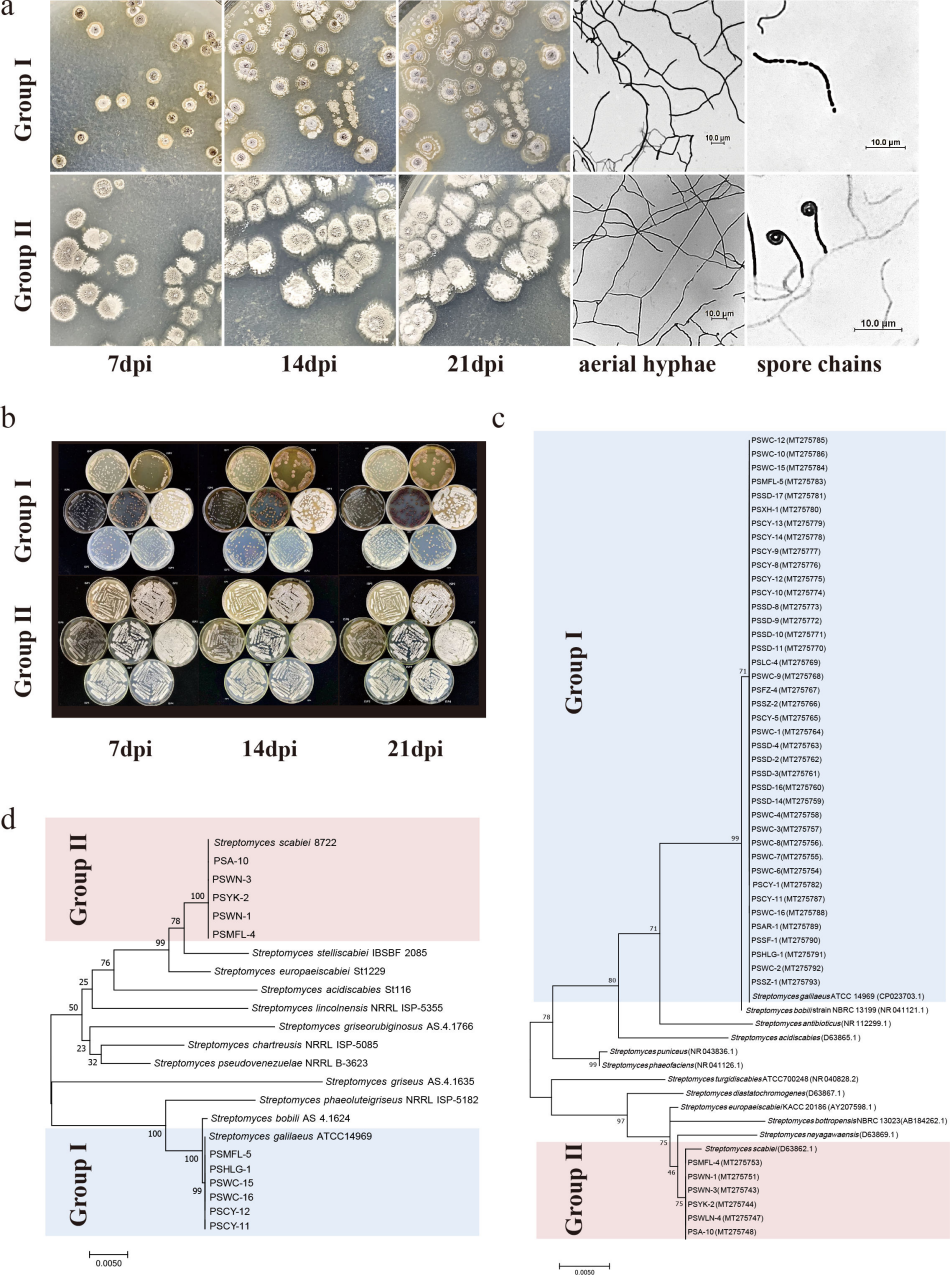
Pathogen identification. Morphology of colony grown on OMA (**a**) and media ISP1–ISP7 (**b**) for 7, 14, and 21 days; aerial hyphae and spore chains under microscope at ×1,000 magnification. Phylogenetic tree of *Streptomyces* spp. isolates and reference species was constructed using the maximum-likelihood method based on concatenated sequences of *16S* rDNA gene. (**c**), *atpD*, *rpoB*, *recA*, and *16S* rDNA (**d**) genes. Bootstrap values were obtained with 1,000 replications. Confidence values of tree nodes are expressed as a percentage of the same tree generated. The length of branches reflects sequence divergence.

The 42 pathogenic strains exhibited two morphological characteristics. Group I consisted of 36 isolates with grayish-white aerial and brown basal hyphae. The edges were round and concentric, and the middle was raised. It produced gray linear spores after 2 weeks. Group II consisted of six isolates with white aerial and basal hyphae. The edges were irregular, and the middle was raised. It produced gray helical spores on the seventh day (Table S2; Fig. 2a). According to morphological observation, group I may belong to the griseorbroviolaceus group; group II may belong to the cinereus group. They grew on all seven media. Group I produced pigments on ISP3, ISP6, and ISP7, while group II did not (Table S3; Fig. 2b).

Two groups could use D-mannitol, I-inositol, raffinose, D-sorbitol, potassium nitrate, ammonium nitrate, sodium nitrate, L-methionine, L-hydroxyproline, and histidine (Table 2). They could hydrolyze starch and liquefy gelatin to produce H_2_S and melanin. They grew on pH 5.0 and 6.0 and 3% NaCl medium and were sensitive to crystal violet, phenol, penicillin, and streptomycin. Group I could not use sucrose and L-rhamnose.

DNA fingerprinting analysis employing BOX-PCR resulted in the identification of two groups. (Fig. S2). The *16S* rDNA gene was amplified for all bacterial isolates (Fig. 2c). Among the isolates, 36 were clustered with *S. galilaeus* (group I), with nucleotide similarity ranging between 99.72% and 100%; 6 were grouped with *S. scabies* (group II), with similarity being 99.73%–100%. To test the accuracy of the above results, we amplified and spliced the *atpD*, *rpoB*, *recA*, and *16S* rDNA genes of 10 representative isolates from two groups for phylogenetic analysis (Fig. 2d). Among the isolates, five were *S. scabies* and five were *S. galilaeus*, consistent with the above results.

### Population genetic analysis reveals high diversity in *S. galilaeus*

**Fig 3:**
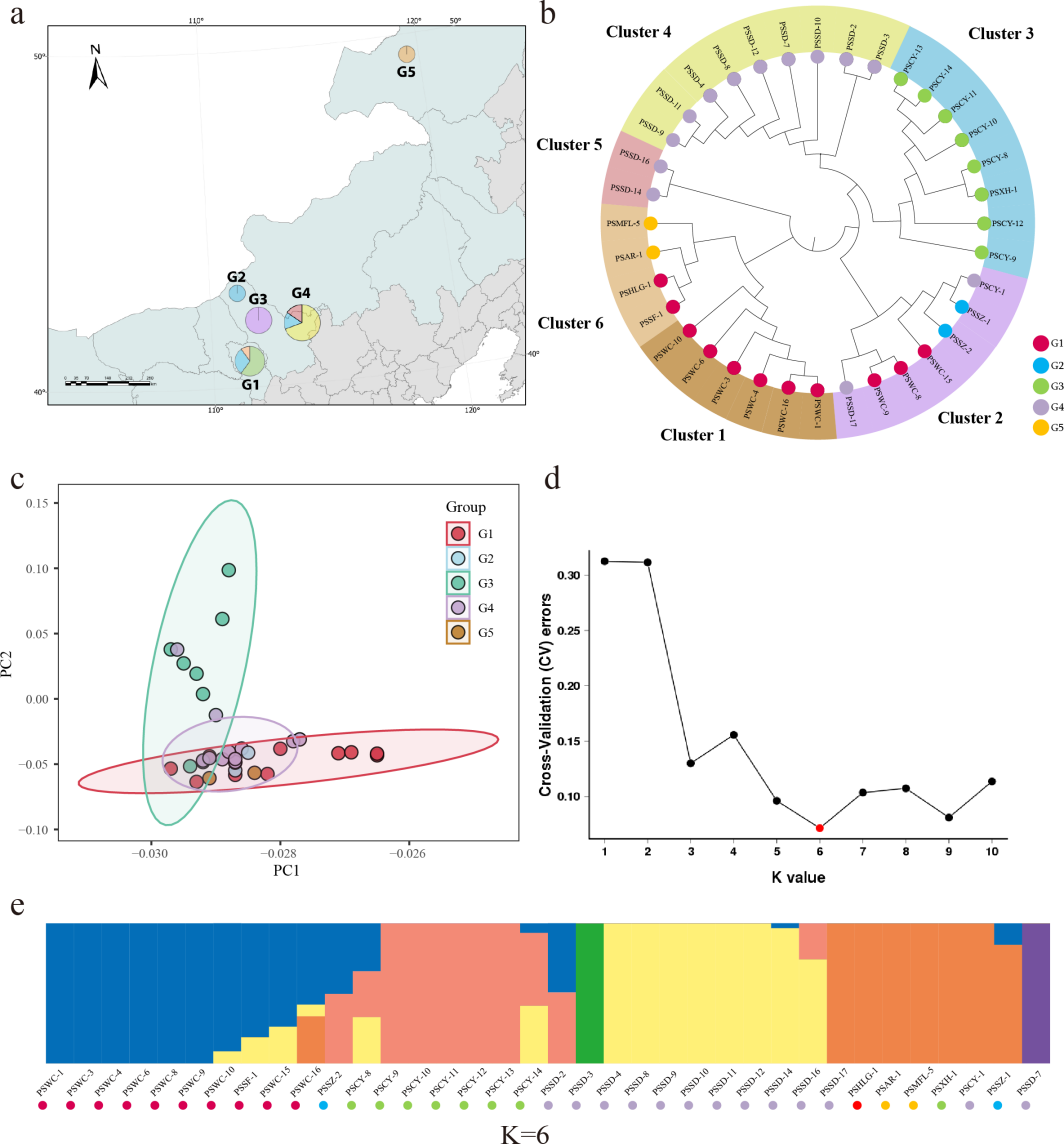
Population structures of *Streptomyces galilaeus* in northern China, based on single nucleotide polymorphism. (**a**) Cluster proportions in different regions. The base map was obtained from the National Catalog Service for Geographic Information (Tianditu) and visualized using ArcGIS Pro. (**b**) Phylogenetic analyses, maximum-likelihood phylogenetic tree was established with 1,000 bootstraps. “G” denotes the geographical group (five regions). (**c**) Principal component analysis of *S. galilaeus* populations. (**d and e**) Population genetic structure of populations inferred by *K* values with the lowest cross-validation error rate of ADMIXTURE.

Individual genomes of 36 *S*. *galilaeus* isolates were resequenced at an average depth of 171× coverage. Reads were aligned to the *S. galilaeus* ATCC 14969 reference genome (GenBank: FN554889.1), achieving an average alignment rate of 94.93%. SNP calling was performed using GATK, followed by stringent filtering (depth ≥10× , QUAL ≥30, excluding flanking complex regions), yielding 32,890 high-quality SNPs for downstream population genetic analyses. PhyloNet analyses sorted the *S. galilaeus* into six major clades (Fig. 3b). Those from clusters 2 and 6 were widely spread throughout most of their natural range, from southwest to northeast (Fig. 3a). Both G4 and G1 contained three clusters, with individuals from clusters 4 and 5 exclusively found in G4, and those from cluster 1 exclusively present in G1. Principal component analysis showed that G1 and G3 were significantly separated, and G4 was similar to other populations.

To infer population structure and clarify the number of ancestors in the population, we conducted population structure analysis using ADMIXTURE. The cross-validation error was minimized at *K* = 6 (Fig. 3c), supporting the model that the tested strains comprise six genetic groups, the structure of which is shown in Fig. 3d. The results show that the G4 population from Shangdu contains all genetic groups and exhibits the highest genetic diversity (Fig. 3e), indicating that there is a large amount of gene flow with other populations.

## DISCUSSION

In our study, we demonstrated that *S. galilaeus* is the predominant causative agent of PCS in northern China, accounting for 85.7% of pathogenic isolates, which contrasts with the global prevalence of *S. scabies* reported in other regions (3). This suggests that local environmental factors, particularly weakly alkaline soil conditions, play a critical role in shaping pathogen composition. We further confirmed that the integration of MLSA with morphological characteristics is essential for accurate species identification, addressing the limitations of *16S* rDNA-based classification in *Streptomyces*. Importantly, this study revealed the genetic diversity and spatial distribution patterns of the species, which provided key clues for tracing the origin of the spread of this pathogen. These findings indicate that region-specific pathogen surveillance and management strategies are necessary for effective control of PCS.

The accurate delineation of species was a cornerstone of this study. Because of subtle variations in *Streptomyces* spp. and isolates, multiple methods should be integrated for identification. For example, *16S* sequencing is known to be inaccurate for *Streptomyces* (35); the MLSA method provided fine resolution in taxonomic analysis (36); and combined with morphology identification, the accuracy of identification results was ensured. The integration of MLSA provided the necessary taxonomic resolution, affirming its value as a standard tool for species-level classification in complex genera. This rigorous approach ensured that subsequent analyses of distribution and virulence were grounded in accurate species assignment.

Common scab is favored by dry and warm soil conditions. Bouchek-Mechiche et al. (37) reported this relationship on *S. turgidiscabies* and *S. europaeiscabiei*. However, the effectiveness is highly dependent on the pathogen’s taxa and environment (6). Soil pH selects *Streptomyces* spp. Lower soil pH suppresses the growth of most species, but *S. acidiscabies* and *S. turgidiscabies* are less affected (38, 39). This means that in an acidic soil, *S. acidiscabies* and *S. turgidiscabies* can be the most abundant species.

The prevalence of pathogen species varies, depending on the location. Soils in northern China were all weakly alkaline (Table S1), suggesting that *S. galilaeus* is more suitable for surviving in alkaline environments. *S. scabies* has been a primary species causing PCS in many countries, including some regions of China (3, 6, 40). *S. acidiscabies* has the highest population in Jilin and Shandong, China, which is possibly due to the acidic condition of the soil. In Yunnan, the southwest region, there are abundant species of *Streptomyces*, and more than 10 possible species have been reported. Among them, *S. enissocaesilis* and *S. anulatus* are predominant species, which are possibly related to the complexity and diversity of climate types and soil conditions (41). This discrepancy strongly suggests that local environmental factors are key drivers of pathogen selection. This underscores the necessity of developing region-specific surveillance strategies based on local pathogen profiles.

Phytotoxins are determinants of the pathogenicity of *Streptomyces* spp. (42). Pathogenicity-related genes include *txtAB*, *tomA*, and *nec1*. We have shown that these genes exist in 42 bacterial isolates, indicating they were pathogenic (11, 43). In this study, the level of virulence was highly related to bacterial species as they each produce various levels and types of toxins (Fig. S3). However, thaxtomins are not the only pathogenic determinant. Natsume et al. (10) found that *S. turgidiscabies* did not produce thaxtomins but rather phytotoxin fridamycin E, which reduces or inhibits sprouting of potato microtubers *in vitro* and produces netted scab lesions on potato tubers. Lapaz et al. (7) found that desmethylmensacarcin from isolate *S. niveiscabiei* is also a toxin involved in pathogenicity. *Streptomyces* spp. 11-1-2 has also been shown to produce other phytotoxic secondary metabolites, such as nigericin and guerdamycin, which also contribute to disease development (44). These results indicate that pathogenic *Streptomyces* have complex and diverse pathogenic strategies, and the virulence mechanism is not a single pathway but the result of multiple toxins and their combinations.

It is noteworthy that lesion type was not strictly species specific, as a single species could induce multiple symptomologies. We have found that superficial lesions were the most common type, followed by pitted lesions. In addition to the above three types, netted scab lesions have also been reported (10). Typical lesions caused by *S. galilaeus* were superficial, while those by *S. scabies* were pitted in our study, which was in agreement with Natsume et al. (45). *S. scabies* produces specific phytotoxins, thaxtomins, and concanamycin; the latter are responsible for pitted lesions. This complexity indicates that symptom manifestation is a multifactorial trait, influenced by the interplay of pathogen virulence factors, host variety, and environmental conditions (46).

Although there were only two major species responsible for PCS in northern China, *S. galilaeus* exhibited a range of genetic variations within the species. A distinct geographical separation in interspecific classification, with clusters 1, 3, 4, and 5 each comprising isolates from the same region. Nevertheless, isolates from three distinct regions were also identified within cluster 2, suggesting gene exchange among these isolates, especially in geographically close regions. Isolates from geographically distant group 6 were grouped, suggesting that species transmission may also be influenced by human activities (47). The isolates from Shangdu G4 were divided into three clusters and contained all six ancestral groups. Isolates PSSD-2, PSSD-3, PSSD-14, and PSSD-16 exhibited close genetic affinity with other samples, indicating that Shangdu may be the origin of *S. galilaeus* transmission in northern China.

The management of PCS presents a significant challenge, primarily due to the soil-borne nature and ecological resilience of pathogenic *Streptomyces* spp. While conventional cultural practices such as crop rotation with non-hosts and the application of green manures can moderate pathogen distribution and spread to some extent, their efficacy is often inconsistent and limited by the complex soil ecology (48). Due to the diversity of pathogenic species and environmental preferences, it is particularly important to carry out targeted management strategies. An example is that since *S. galilaeus* has been recognized as a predominant species of pathogen in this region, *Bacillus velezensis* strain 8-4 and *Streptomyces* spp. strain PBSH9 have been applied to control the disease, which has shown a promising result (31, 49). In addition to precise control, comprehensive management, such as long-term soil health construction and host resistance, improvement is essential.

In conclusion, our work provides a comprehensive analysis of the PCS pathosystem in northern China, revealing *S. galilaeus* as the dominant pathogen and elucidating its population structure. The growing problem of PCS in China, especially in northern China, makes developing an effective management strategy a priority. The effectiveness of PCS management, whether through cultural practices, biocontrol, or resistant cultivars, is highly dependent on the target pathogen species and local environment. Therefore, the insights gained here into the identity, distribution, and genetics of the predominant pathogen are fundamental for designing targeted and sustainable management strategies against this important disease.

## Data Availability

The original sequencing data of this study were deposited in the Sequence Read Archive under BioProject accession number PRJNA1357885, with individual sample accession numbers SRR35954562–SRR35954597. All *16S* rDNAs have been deposited in GenBank under accession numbers MT275743–MT275793. Scripts employed in the computational analyses are available at https://github.com/liyuchen3303/SNP.git.

## SUPPLEMENTAL MATERIAL

Supplemental material spectrum.03050-25-s0001.docx Tables S1 to S3; Fig. S1 to S5.

ASM does not own the copyrights to Supplemental Material that may be linked to, or accessed through, an article. The authors have granted ASM a non-exclusive, world-wide license to publish the Supplemental Material files. Please contact the corresponding author directly for reuse.
