# Supplementary material for: Genetic diversity of Streptomyces species causing potato common scab in northern China
Source: Microbiol Spectr. 2026 Mar 19;14(4):e03050-25. doi: 10.1128/spectrum.03050-25 (PMC13055265; doi:10.1128/spectrum.03050-25)
Supplement: Supplemental material — Tables S1 to S3; Fig. S1 to S5. [file spectrum.03050-25-s0001.docx]

**Supplemental materials**

Table S1. Collection site samples and soil characteristics

| City/county | Cultivar | | Soil type* | Soil texture* | Soil pH* |
| --- | --- | --- | --- | --- | --- |
| Hailar | Maifulao, Xingjia No.2 | Commercial potato | Chestnut soil | loam | 7.8 |
| Yakeshi | Favorita | Commercial potato | Chernozem | loam | 7.8 |
| Zhalantun | Atlantic | Commercial potato | Dark brown soil | loam | 7.6 |
| Arong Qi | Favorita | Commercial potato | Dark brown soil | loam | 7.5 |
| Aershan | 1-1-9 improved varieties | Commercial potato | Grey forest soil | loam | 7.6 |
| Keshiketeng | Jizhangshu No.12 | Commercial potato | Chestnut soil | loam | 8 |
| Wengniute | Jizhangshu No.12 | Commercial potato | Chestnut soil | loam | 7.7 |
| Liangcheng | Kexin No.1, Jizhangshu No.12 | Commercial potato | Chestnut soil | loam | 7.5 |
| Siziwangqi | Kexin No.1 | Commercial potato | Chestnut soil | loam | 7.9 |
| Fengzhen | Kexin No.1 | Commercial potato | Chestnut soil | loam | 7.8 |
| Chahar Right Middle | Kexin No.1 | Minituber | Chestnut soil | loam | 8.0 |
| Shangdu | Zaodabai, Zihuabai, Hoqihong | Commercial potato | Chestnut soil | loam | 7.7 |
| Xinghe | Shepody | Commercial potato | Chestnut soil | loam | 7.8 |
| Huade | Kexin No.1 | Commercial potato | Chestnut soil | loam | 7.7 |
| Chahar Right Back | Shepody | Commercial potato | Chestnut soil | loam | 7.5 |
| Jining | Favorita | Minituber | Chestnut soil | loam | 7.9 |
| Wuchuan | Kexin No.1 | Commercial potato | Grey cinnamon soil | Sandy loam | 7.4 |
| Hollinger | Kexin No.1 | Commercial potato | Grey cinnamon soil | Sandy loam | 8.2 |

^*^Data from Inner Mongolia Mengcao Life Community Big Data Co., Ltd.

Table S2. Morphological characteristics of bacterial isolates

| Isolate | Aerial mycelium color | Spore chain and spore color | Substrate mycelium color | Pigment | Colony morphology | Spore chain morphology |
| --- | --- | --- | --- | --- | --- | --- |
| GroupⅠ | GW^z^ | G | BR | BR | Round, concentric | Linear |
| GroupⅡ | W | G | W | No | Irregular edges, raised middle | Helical |

^z^ W, white; BR, brown; G, gray; GW, grayish white.

Table S3. Culture characteristics of pathogenic bacteria on different media

|  |  | GroupⅠ | | | GroupⅡ | | |
| --- | --- | --- | --- | --- | --- | --- | --- |
| ISP medium |  | 7d | 14d | 21d | 7d | 14d | 21d |
| 1 | Substrate mycelium | W | W | W | W | W | W |
|  | Aerial mycelium | W | W | W | W | W | W |
|  | Soluble pigment | NO | NO | NO | NO | NO | NO |
| 2 | Substrate mycelium | W | LR | LR | W | W | W |
|  | Aerial mycelium | W | W | W | GW | GW | GW |
|  | Soluble pigment | NO | NO | NO | NO | NO | NO |
| 3 | Substrate mycelium | BR | BR | BR | W | W | W |
|  | Aerial mycelium | GW | GW | GW | W | W | W |
|  | Soluble pigment | BR | BR | BR | NO | NO | NO |
| 4 | Substrate mycelium | W | W | W | W | W | W |
|  | Aerial mycelium | W | W | W | W | W | W |
|  | Soluble pigment | NO | NO | NO | NO | NO | NO |
| 5 | Substrate mycelium | W | W | W | W | W | W |
|  | Aerial mycelium | P | P | P | W | W | W |
|  | Soluble pigment | NO | NO | NO | NO | NO | NO |
| 6 | Substrate mycelium | G | G | G | G | G | G |
|  | Aerial mycelium | GW | GW | GW | G | G | G |
|  | Soluble pigment | B | B | B | NO | NO | NO |
| 7 | Substrate mycelium | P | LR | DR | W | W | W |
|  | Aerial mycelium | P | LR | DR | W | W | W |
|  | Soluble pigment | B | B | B | NO | NO | NO |

W, white; B, black; G, gray; P, pink;GW, grayish white; BR, brown; DR, deep red; and LR, light red.


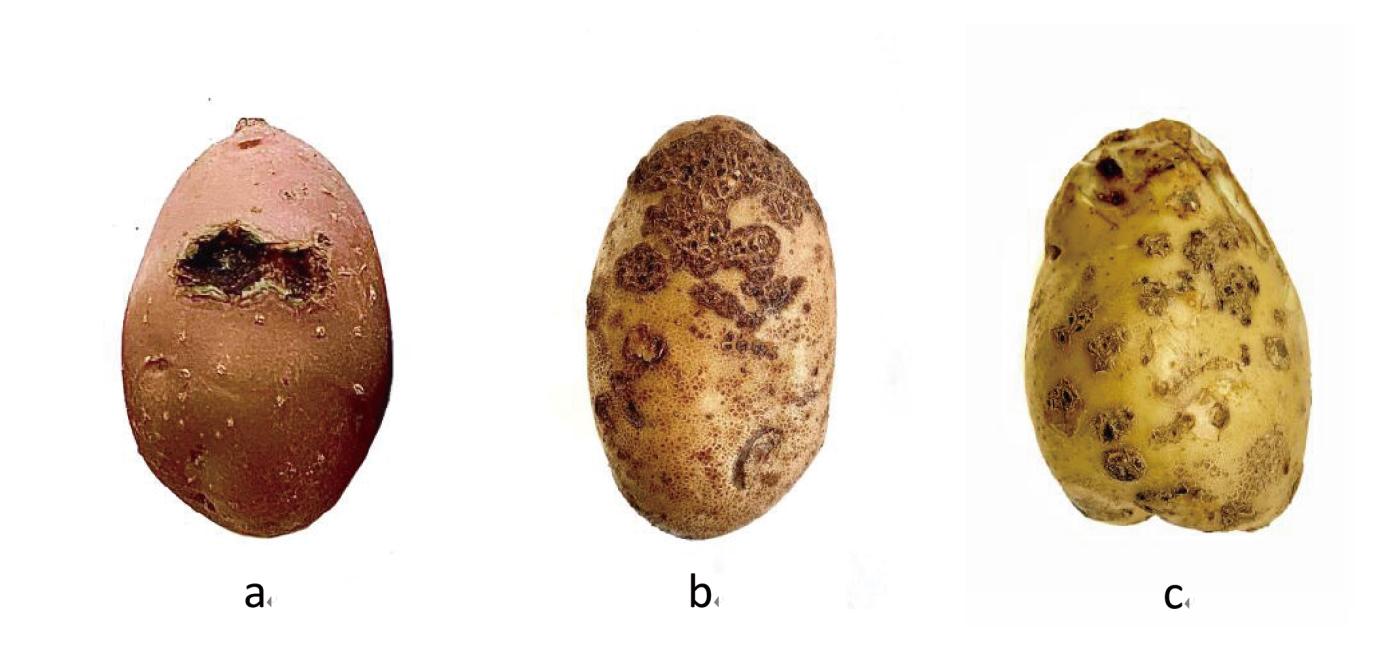
Figure S1. Symptoms of potato common scab, including (a) pitted lesion, (b) raised lesion, and (c) superficial lesion.
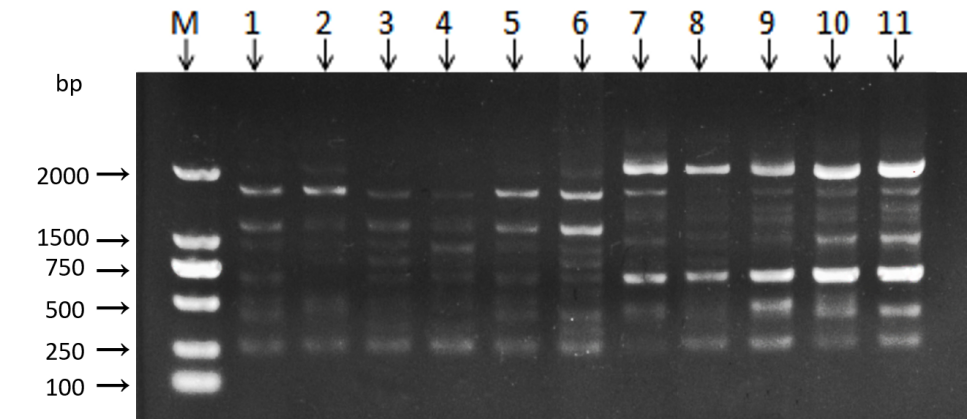
Figure S2. Gel electrophoresis based on repetitive sequence polymerase chain reaction of 11 *Streptomyces* spp. isolates. Lanes included M: DL2000 DNA marker, from 1 to 11: isolates

PSA-10, PSWN-1, PSWN-3, PSYK-4, PSWLN-4, PSMFL-4, PSWC-8, PSWC-9, PSWC-10, PSWC-12, and PSWC-1.


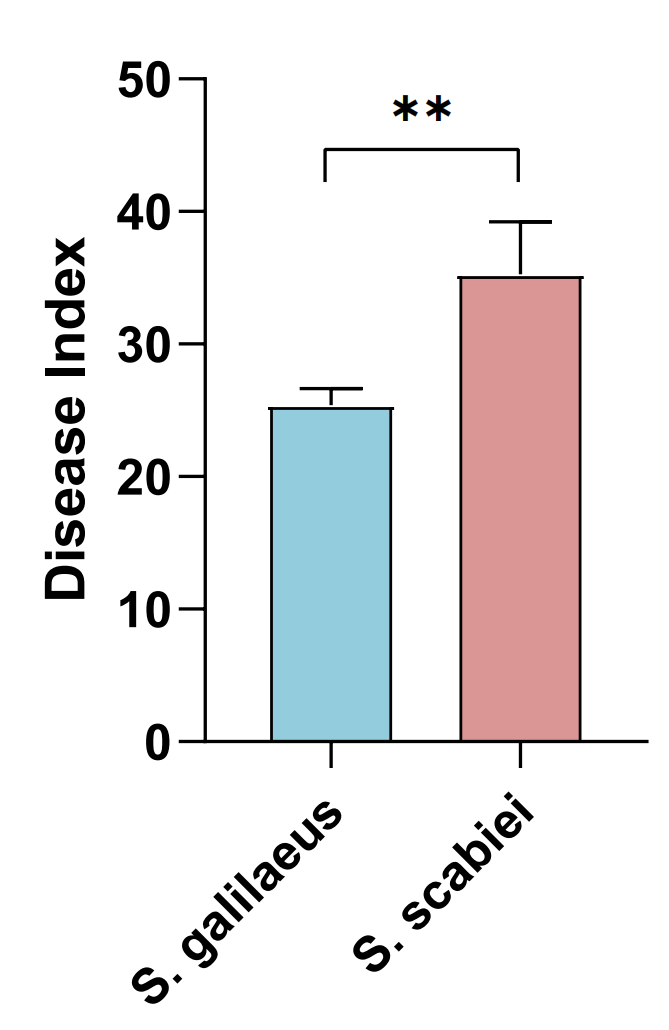
Figure S3. Potted Disease index of *S. galilaeus,* and *S. scabies*.


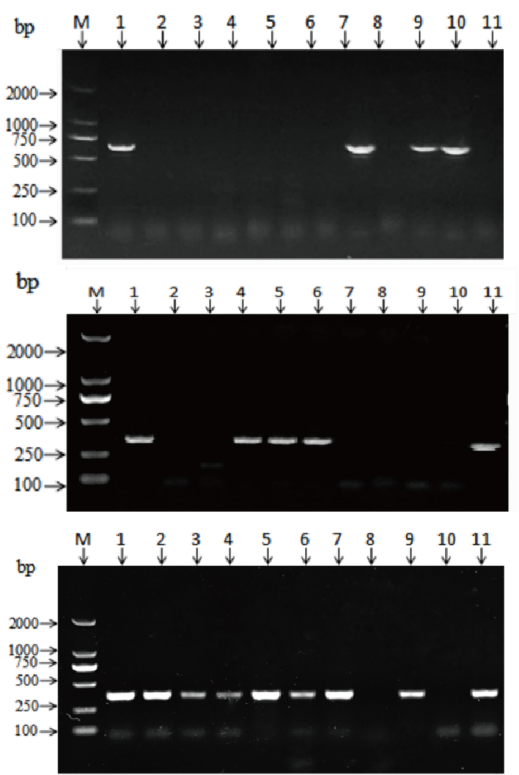
Figure S4. Gel electrophoresis of polymerase chain reaction products of PAI genes. Top panel, *nec1* gene of (from 1 to 11) PSLC-4, PSJH-1, PSZF-2, PSCF-1, PSMF-9, PSMF-11, PSZF-1, PSWN-1, PSMF-10, PSAR-1 and PSWN-3. Middle panel: *txtAB* gene of (from 1 to 11) PSLC-4, PSZF-2, PSCF-1, PSWN-1, PSAR-1, PSWN-3, PSMF-9, PSMF-10, PSMF-11, PSZF-1; and PSMFL-5. Bottom panel: *tomA* gene of (from 1 to 11) PSLC-4, PSWC-2, PSWC-3, PSWN-1, PSAR-1, PSWN-3, PSWC-4, PSMF-10, PSWC-5, PSZF-1; and PSMFL-5.


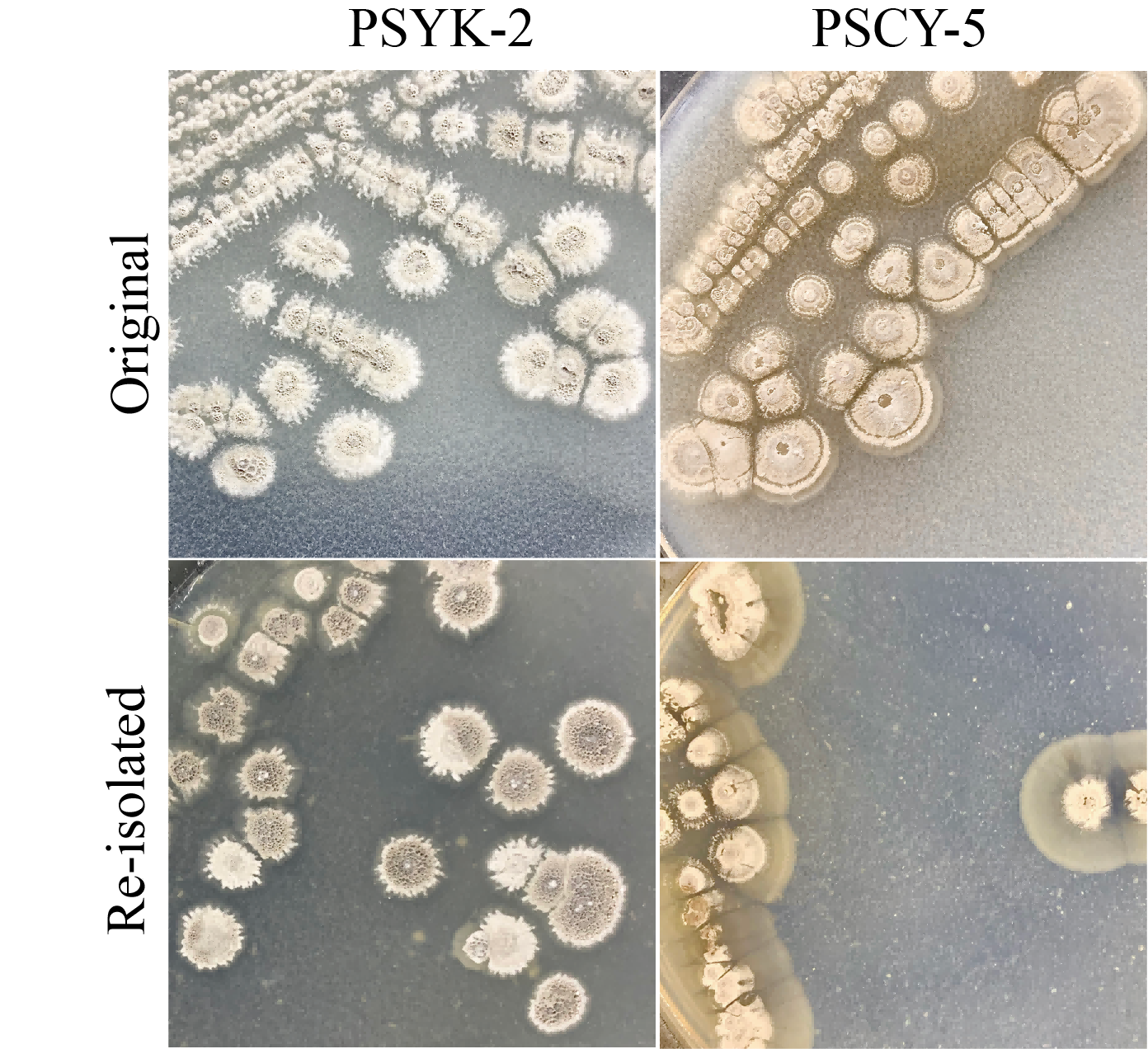
Figure S5. Morphological comparison of reisolated and original strains.
